# Kidney Disease in Diabetes Mellitus: Cross-Linking between Hyperglycemia, Redox Imbalance and Inflammation

**DOI:** 10.5935/abc.20190077

**Published:** 2019-05

**Authors:** Rayne Gomes Amorim, Glaucevane da Silva Guedes, Sandra Mary de Lima Vasconcelos, Juliana Célia de Farias Santos

**Affiliations:** Universidade Federal de Alagoas - Faculdade de Nutrição, Maceió, AL - Brazil

**Keywords:** Diabetes Mellitus/complications, Kidney, Diseases, Oxidation-Reduction, Inflammation, Oxidative Stress, Renal Dialysis

## Abstract

Chronic hyperglycemia is the key point of macro- and microvascular complications associated with diabetes mellitus. Excess glucose is responsible for inducing redox imbalance and both systemic and intrarenal inflammation, playing a critical role in the pathogenesis of diabetic kidney disease, which is currently the leading cause of dialysis in the world. The pathogenesis of the disease is complex, multifactorial and not fully elucidated; many factors and mechanisms are involved in the development, progression and clinical outcomes of the disease. Despite the disparate mechanisms involved in renal damage related to diabetes mellitus, the metabolic mechanisms involving oxidative/inflammatory pathways are widely accepted. The is clear evidence that a chronic hyperglycemic state triggers oxidative stress and inflammation mediated by altered metabolic pathways in a self-perpetuating cycle, promoting progression of cell injury and of end-stage renal disease. The present study presents an update on metabolic pathways that involve redox imbalance and inflammation induced by chronic exposure to hyperglycemia in the pathogenesis of diabetic kidney disease.

## Introduction

Diabetic kidney disease (DKD) is a devastating outcome of diabetes mellitus (DM), responsible for high morbidity and overall mortality. It is clinically characterized by persistent renal dysfunction for a period equal to or longer than three months, marked by urinary excretion of albumin >30 mg/24 h or an albumin/creatinine ratio (ACR) ≥ 30 mg/g or glomerular filtration rate (GFR) < 60 mL/min/1.73 m after a hyperfiltration phase, in addition to structural abnormalities (e.g. diabetic glomerulosclerosis) in individuals with previous diagnosis of DM.^1,2^

It is estimated that approximately 425 million people have DM in the world, with a projected increase by 48% to the yar of 2045. Approximately 12.5 million people are diagnosed with DM in Brazil, which occupies the third position in number of individuals with DM in the world in 2017.^3^ Nearly 90% of DM patients develop microvascular and macrovascular complications; DKD is considered one of the most severe clinical outcomes, affecting 20-40% of the patients, most of them type 2 DM patients.^1^ DKD is currently the main cause of dialysis in developed countries, the second main cause in Brazil.^4-6^

DKD is a progressive and irreversible condition, whose pathogenesis has been associated with functional and structural changes of renal cells in response to metabolic stress induced by excessive glucose inflow, by means of activation of specific metabolic pathways linked to redox imbalance and inflammation.^7^

Although many classical mechanisms involved in the development and progression of DKD have been described, new molecular and epigenetic pathways have been suggested to be responsible for the early kidney functional loss and DKD-related complications.^8^

In this review we discuss current knowledge of metabolic pathways involving redox imbalance and inflammation induced by chronic exposure to hyperglycemia in the pathogenesis of DKD, aiming to propose new paradigms.

### Pathophysiology of DKD induced by hyperglycemia: new paradigms

DKD is a chronic metabolic disease in which hyperglycemia causes dysfunction of renal and vascular cells. The pathophysiology of DKD and the consequent end-stage renal disease requiring dialysis is caused by a chronic hyperglycemic state that leads to activation and changes of metabolic pathways and hemodynamic dysfunction. Some of these changes occur in an integrated way, leading to several other changes. Although diabetic hyperglycemia is an important but not crucial factor for the development of glomerular lesions in DKD, we will describe metabolic changes induced by intermittent and chronic exposure to hyperglycemia. The following topics will be discussed: glucose auto-oxidation, polyol and hexamine pathways, formation of advanced glycation end-products (AGEs), synthesis of nicotinamide adenine dinucleotide phosphate (NADPH) oxidase (NOX), protein kinase C (PKC) activation, and abnormal activity of angiotensin II (Ang II).^9,10^

### Glucose uptake by diabetic kidney cells

Hyperglycemia is the main clinical manifestation of DM, the main driving force for the development of chronic complications of the disease, including DKD. It is caused by two main mechanisms: the first involves dysfunction and apoptosis of pancreatic beta cells caused by an autoimmune abnormality (type 1 DM), and the second results from an overstimulation of insulin synthesis and secretion in the presence of insulin resistance (IR), mostly associated with overweight/obesity, which characterizes type 2 DM.^11^

In the context of obesity, which is common in patients at risk of type 2 DM, IR results from increased levels of free fatty acids (FFAs), and the FFA by-products pro-inflammatory cytokines and diacylglycerols (DAG), that inhibit phosphorylation of the insulin receptor substrate 1 (IRS-1) in phosphorylation domains (serine/threonine), preventing the propagation of signals to the translocation of the glucose transporter-4 (GLUT4) translocation to the plasma membrane. This affects the interaction between insulin and its receptor, leading to decreased glucose uptake by insulin-dependent cells, and ultimately hyperglycemia and hyperinsulinemia.^12,13^

In additional, in obese diabetic individuals, excessive accumulation of fat causes stress of adipocytes by hyperplasia and hypertrophy, leading to hypoxia and subclinical inflammation, increased macrophage infiltration and release of pro-inflammatory cytokines - tumor necrosis factor alpha (TNF-α), interleukin-6 (IL-6) and interleukin-1 (IL-1) - which, in turn, aggravates IR.^12,14^ TNF-α stimulates the secretion of other cytokines and chemokines and directly activates the transcription factor-kappa B (NF-kappa B), thereby leading to maintenance of chronic hyperglycemia, by affecting glucose uptake and promoting IR.^15^

In attempt to reestablish homeostasis, glucose uptake occurs in cells not dependent on GLUT4, and hence, non-insulin-dependent, such as renal cells, that have GLUT 1 and GLU2 as glucose transporters. These glucose carriers do not regulate glucose entry into cells, leading to glucotoxicity. In this situation, there is elevated expression of these transporters, leading to increased entry of glucose into renal cells, as occurs with the high-affinity glucose transporter GLUT 2, stimulated by hyperglycemia, and the sodium-glucose co-transporter (SGLT) 1 and SGLT 2, responsible for tubular reabsorption.^16,17^ Thus, in DM patients, glucose reabsorption in the proximal tubule is increased, contributing to hyperglycemia and, consequently, hyperfiltration.^18^

### Generation of reactive oxygen and nitrogen species (RONS) induced by hyperglycemia

Glucotoxicity is caused by an inability of the cells to compensate for the increased glucose uptake in case of IR/hyperglycemia, as in DM. Increased stimulation of glucose oxidation pathways in non-insulin-dependent cells leads to the activation of alternative pathways, increased production of RONS and oxidative stress (OS) in hyperglycemic state.^19^

### Glucose auto-oxidation

In glycemic homeostasis and in the absence of oxygen, glycolysis is the primary energy source in the cells. By-products of these reactions are coenzymes responsible for the uptake of high energy electrons, released during oxidation-reduction (redox) reactions, that participate in additional energy pathways.^20^ The synthesis of substrates by glycolysis activates two other energy pathways: the tricarboxylic acid cycle (or the Krebs cycle) and the electron transport chain (ETC) (or the oxidative phosphorylation) in the mitochondria through protein complexes.^20-22^

In DM, the hyperglycemic state promotes the overactivation of the three main energy pathways previously described. The increased stimulation of glycolysis and Krebs cycle result in elevated production of reduced flavin adenine dinucleotide (FADH2) and reduced nicotinamide adenine dinucleotide (NADH), fed into the ETC.^23^

**Figure 1:**
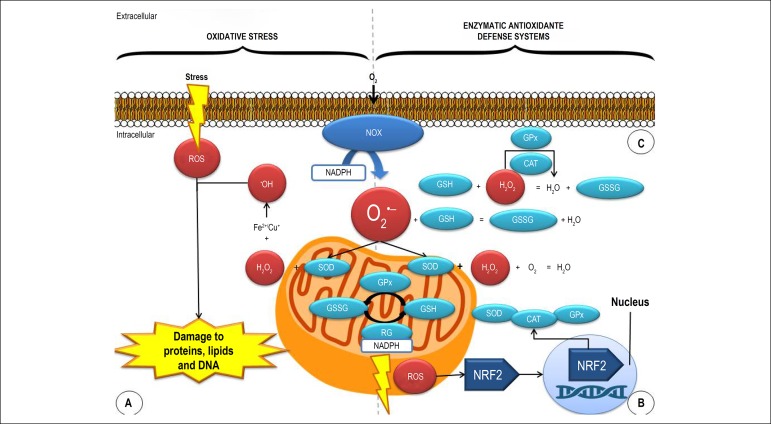
Oxidative stress and enzymatic antioxidant defense system in diabetic renal cells. CAT: catalase; ROS: reactive oxygen species; GPx: glutathione peroxidase; GSH: glutathione; GSSG: oxidized glutathione; RG: reduced glutathione; H_2_O_2_: hydrogen peroxide; NRF2: nuclear erythroid 2-related factor 2; O_2_: molecular oxygen; NOX: NADPH oxidase; O_2_^•-^: superoxide anion radical; ^•^OH: hydroxyl radical; SOD: superoxide dismutase. Adapted from Bhargava.

The ETC is a source of reactive oxygen species (ROS), especially in renal cells, which have a large number of mitochondria.^24^ In diabetic kidney cells, highly stimulated, hyperpolarized mitochondria with high redox potential, produce increased levels of adenosine triphosphate (ATP) and superoxide anion (O_2_^-•^) through the complexes I and II. O2-• originates both radical and non-radical RONS, including hydrogen peroxide (H2O2), the hydroxyl radical (•OH) and the ONOO^-^, which may be involved in the genesis of the lesions (Figure 1).^25,26^

In the kidney of diabetic or diabetic/obese individuals, mitochondrial energy, altered by hyperglycemia and hyperlipidemia, causes mitochondrial dysfunction and excess ROS, which are harmful to mitochondrial DNA (mtDNA) by inhibiting the mammalian target of rapamycin complex 1 (mTORC1) and the AMP-activated protein kinase (AMPK). These changes alter the activation of the peroxisome proliferator-activated receptor gamma coactivator 1-alpha (PGC-1α), and thereby affect mitochondrial biogenesis, by increasing mitochondrial fission and the synthesis of defective mitochondria. This can lead to impairment of the ECT functions, with reduced synthesis of ATP, leading to renal cell lesion and apoptosis.^27^

In addition, increased glycolysis causes hyperactivation of polyol and hexosamine metabolic pathways, and increases the synthesis of AGEs and activation of PKC. This also result in decreased ATP levels, contributing to mitochondrial dysfunction and fragmentation.^28^

### Polyol pathway

One of the consequences of the increased glucose uptake by the cells is the increment in the NADPH-dependent conversion of glucose into sorbitol by aldose reductase. Sorbitol is then converted to fructose, by nicotinamide adenine dinucleotide (NAD).^29,30^ NADPH is an important cofactor for regeneration of the antioxidant glutathione (GSH). Therefore, in light of increased aldose reductase activity, the low availability of NADPH affects the antioxidant defense, resulting in redox imbalance.^31^

The increase in O2•- inhibits the activity of glyceraldehyde 3-phosphate dehydrogenase (GAPDH), which, in turn, inhibits glycolysis and activates alternative pathways. The increase in the NADH/NAD ratio increases the production of DAG, which activates PKC. Fructose, an end-product of the pathway, has been recently related to kidney injury markers.^20^

### Protein kinase C (PKC)

**Figure 2:**
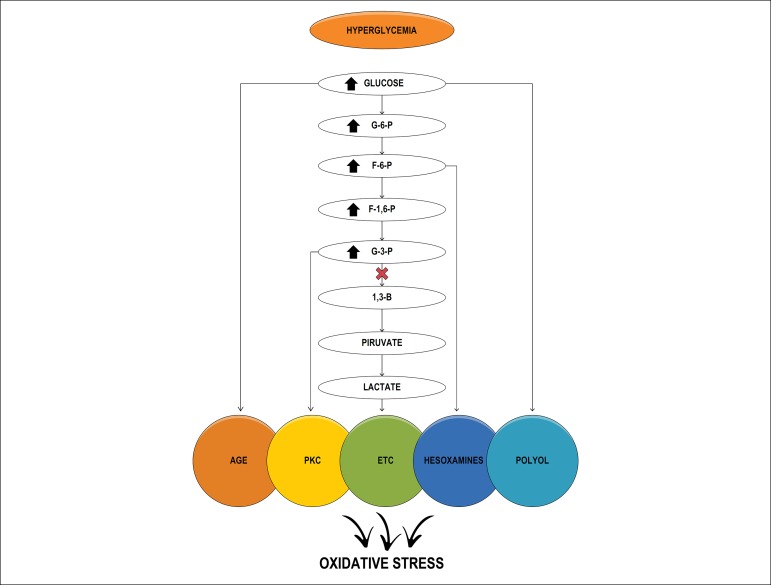
Schematic representation of the pathways preceding glycolysis and induction of oxidative stress. ETC: electron transport chain; 1,3-BPG: bisphosphoglycerate; G6P: glucose 6-phosphate; G3P: glyceraldehyde-3-phosphate; GAPDH: glyceraldehyde-3-phosphate dehydrogenase; F6P: fructose-6-phosphate; F-1,6-P: fructose-1,6-phosphate; PKC: protein kinase C; AGE; advanced glycation end-products

Both hyperglycemia and increased stimulation of glycolysis by-products increase the synthesis of glyceraldehyde-3-phosphate and its conversion into dihydroxyacetone, and thereby promote the synthesis of DAG, a PKC-activating factor (Figure 2).^31^

In renal cells, increased PKC stimulates several mechanisms involved in the development of kidney injury. The induction and activation of endothelial nitric oxide synthase (eNOS) by PKC increases the availability of nitric oxide (NO) in diabetic kidney in the first stages of DKD.^32^ Increased NO contributes to elevation of prostaglandin E1 levels, Ang II activity, and activation of the vascular endothelial growth factor (VEGF), resulting in increased permeability, endothelial dysfunction, glomerular hyperfiltration and albuminuria.^33,34^

In prolonged diabetes, persistent hyperglycemia reduces the levels of tetrahydrobiopterin (BH4), an eNOS cofactor, with proportional reduction in NO synthesis in vascular endothelium, leading to vasoconstriction and glomerular and systemic hypertension.^34^

Hyperglycemia-related endothelial damage is caused by a nitroso-redox imbalance, by increased RONs (resulting from the interaction between O2•- and NO, which leads to increased ONOO- and decreased vascular NO), leading to endothelial dysfunction and DKD progression.^35^

Increased expression of PKC leads to activation of the transforming growth factor-beta (TGF-β) and the plasminogen activator inhibitor-1 (PAI-1), resulting in increased deposition of fibronectin, collagen types I and IV and extracellular matrix deposition, and consequently, renal hypertrophy, glomerulosclerosis and renal fibrosis.^30^

### Hexosamines

The hyperfunction of this pathway, stimulated by hyperglycemia, promotes the conversion of fructose 6-phosphate, and the synthesis of uridine diphosphate N-acetylglucosamine (UDP-GlcNAc) as end product, which is *O*-glycosylated into N-acetylglucosamine (*O*-GlcNAc) by the *O*-GlcNAc transferase.^29,30^

The excess of *O*-GlcNAc is responsible for stimulating and modifying cell protein. In DKD, changes in genetic expression increase TNF-α transcription, thereby inducing renal damage via OS, and overproduction of extracellular matrix proteins.^20,29,36^

### Advanced glycation end products (AGEs)

AGEs are uremic toxins and their involvement in the development of renal damage may be partially explained by increased endogenous synthesis resulting from hyperglycemia, diet and insufficient clearance of these products due to reduced GFR.^37^

AGEs are formed through non-enzymatic amino-carbonyl reactions, or Maillard reaction between the carbonyl group of glucose, fructose, galactose and ribose, or intermediates of glucose metabolism (glucose-6-phosphate, fructose-6-phosphate, ribose-5-phosphate, deoxyribose-5-phosphate and glyceraldehyde), with an amine group and other molecules, to form a reversible Schiff base, and subsequently, Amadori products, which are initial products of the Maillard reaction.^13^ Synthesis of the Amadori products is accelerated in hyperglycemic conditions, and these compounds are highly reactive with amine groups and metal ions through glycoxidation of biological molecules, forming glyoxal (GO), methylglyoxal (MGO), and malondialdehyde (MDA).^38,39^

After AGEs' metabolism and removal from the tissues, the low molecular weight, soluble peptides and the second generation AGEs need to be excreted in the urine. Second generation AGEs can be highly reactive products, but their effects are limited by renal excretion. However, in renal failure, AGEs excretion is impaired, contributing to the increased levels in serum and tissues.^38^

Such increase in the endogenous pool of AGEs causes direct damage to the cells by interaction of extracellular proteins with cellular components (proteins, carbohydrates, lipids and nucleotides), affecting cellular structure and functions. These modified proteins have decreased enzymatic hydrolysis, resulting in excessive accumulation of extracellular matrix proteins, glomerulosclerosis, and consequently, renal fibrosis.^34^

In addition to direct extra- and intracellular damage, AGEs interact with their transmembrane receptor - the receptor for advanced glycation end products (RAGE), which are expressed in many types of renal and inflammatory cells.^40^ Following the substrate/receptor interaction, a cascade of reactions inside the cell is initiated. These reactions regulate the transcription of proteins, adhesion molecules and proinflammatory cytokines, such as IL-1, IL-6 e TNF-α, mediated by the activation of macrophages via NF-κB, exacerbating subclinical tissue inflammation associated with DM in DKD.^41,42^

The AGEs/RAGEs interaction is associated with increased production of RONs; it contributes to the OS by direct activation of NOX (by mitochondrial activation through the RAGEs in renal cells and infiltrated immune cells). Also, AGs reduces the expression of eNOS and increases the expression of inducible NOS (iNOS), triggering OS by increased ONOO^-^ production and reducing NO availability. Consequently endothelial dysfunction occurs by synthesis of vascular cell adhesion molecule 1 (VCAM-1), intercellular adhesion molecule 1 (ICAM-1), monocyte chemotactic protein-1 (MCP-1) and TGF-β.^38,39^

### NADPH-oxidases

The NOX family of NADPH oxidases is an important source of ROS in DM. There are seven different NOX isoforms: NOX1, NOX2, NOX3, NOX4 (formerly known as "renox" due to its high expression in renal tissue), NOX5 and the dual oxidases 1 and 2 (DUOX1 and DUOX2, respectively).^9^ NADPH oxidases are transmembrane proteins responsible for transferring electrons from cytosolic NADPH to the O_2_, which is reduced to O_2_^•^, thereby perpetuating the oxidative stress state in renal cells.^9,25^

NOX-derived ROS regulate physiological processes in the kidneys. However, they are upregulated in hyperglycemic renal cells, and abnormally activated by AGEs, PKC, TGFβ and Ang II, resulting in O_2_^•-^ overproduction and accumulation. O_2_^•-^ acts as an important mediator of redox imbalance and damage to different kidney cell components.^9,43^

### Angiotensin II

Chronic hyperglycemia in DM induces increased synthesis of Ang II and its receptors by glomerular and mesangial cells, and podocytes. It increases the expression of renin and angiotensinogen in mesangial cells, elevating intrarenal angiotensin levels. This mechanism is exacerbated by ROS accumulation in adipose tissue, where Ang II is produced.^34,44^

Elevations of Ang II contribute to abnormal activation of the renin-angiotensin-aldosterone system (RAAS), aggravating mechanical damages induced by systemic and intraglomerular hypertension in the kidney. Additional effects of Ang II include direct mediation in RON production, early hyperplasia and late hypertrophy of renal cells, by stimulation of TGF-β, IL-6 and MCP-1, and activation and upregulation of NF-κB.^45^

### Hemodynamic changes in diabetic kidney damage

Early DKD is marked by changes in renal hemodynamics caused by hyperglycemia.^46^ The initial hemodynamic events are characterized by glomerular hyperperfusion, hypertension and hyperfiltration, and responsible for functional and structural changes in the glomeruli, resulting in albuminuria, increase followed by decrease of GFR, glomerular hypertrophy, mesangial expansion, podocyte injury, glomerulosclerosis and renal fibrosis, and natural history DKD.^47,48^

Hypertension commonly precedes DKD, especially in DM2. However, persistent metabolic disturbances cause sustained hypertension, and dysregulation of pressure levels, inducing and/or aggravating diabetic kidney injury.^45^ The mechanism of hypertension in DKD is complex, multifactorial, and involves altered sodium regulation, such as renal tubular reabsorption of sodium, abnormal activation of the RAAS and of sympathetic nervous system (SNS), endothelial cell dysfunction, and increased OS. These processes mediate vasoconstriction and increase extracellular volume with consequent increase in blood pressure.^49,50^

Among hemodynamic factors that contribute to hypertension and renal hyperfiltration, RAAS has been the most widely accepted for the development of DKD, and its blockade has shown to delay the progression of the disease.^51^ Mechanical stress on vascular wall induced by hypertension, hyperglycemia, inflammation and ROS considerably increase Ang II production in renal cells and contribute to RAAS hyperactivation.^45,51^ This in turn, contributes to systemic and renal vascular vasoconstriction, and renal reabsorption of sodium via interaction with Ang II receptor type 1 (AT1) and aldosterone release, leading to elevations in blood pressure, intraglomerular pressure and renal damage.^52^

The effects of Ang II on redox imbalance (additional effects of Ang II on inflammation and redox imbalance, and important factors in the pathophysiology of DKD are described above) via production of O2^•-^ by local NOXs induce endothelial dysfunction (due to an imbalance between vasoconstrictor and vasodilator factors).^53^ In response to increased ROS, there is a reduction in the synthesis of NO, a potent vasodilator that interacts with the substrate BH4, reducing eNOS activity. Besides, there is a direct effect of the O2^•-^ on reducing NO and ONOO^-^, and reducing NO availability, leading to sustained vasoconstriction.^50^

The actions of Ang II, in addition to endothelial dysfunction, vasoconstriction and vascular resistance, induced by the OS, result in elevations in the pressure of afferent arterioles, which, in turn, cause an increase in systemic blood pressure, glomerular hyperperfusion and hyperfiltration, and proteinuria, leading to progressive DKD.^50^

In addition, sodium-hydrogen exchangers (NHEs) play an important role on renal and systemic hemodynamics in DKD. NHEs are expressed in different types of renal cells and regulate sodium (Na^+^) and hydrogen (H^+^) transport, essential for different cell functions, including maintenance of intracellular pH, fluid volume and cell survival.^54^ In the kidneys, particularly in tubular cells and macula densa cells, NHE isoforms 1, 2 and 3 (NHE1, NHE2 and NHE3, respectively) play an important role in the pathogenesis of DKD, by inducing intraglomerular hypertension and mesangial proliferation, and by promoting of inhibiting programmed cell death (apoptotic factors), contributing to renal fibrosis.^55^

In macula densa cells, the NHE2 receptors are involved in the regulation of renin and salt sensors. The suggested mechanism is that cell shrinkage (induced by hypertonicity), together with Ang II, is the renin-release signal, leading to the overexpression of the RAAS and increase in intraglomerular pressure. This would activate the signaling pathway that results in increased expression of the NHE receptors in renal cells (promoting a vicious circle).^56^ Also, salt excess, induced by NHE in the macula densa, causes an increase in intracellular pH and cell depolarization, leading to activation of ROS synthesis by the NOX enzymes.^57^

NHEs are targets of many drug therapies, including inhibitors of the RAAS and of SGLTs, which are involved in NHE blockade in the kidneys, contributing to reduction of intraglomerular pressure, and of proliferative and fibrotic processes.^56^

Blockade of the RAAS and Ang II with angiotensin-converting enzyme inhibitors and Ang II receptor blockers (either combined or alone), have shown to be effective in reducing proteinuria and delaying the progression of DKD by their hemodynamic/anti-hypertensive, anti-inflammatory and antifibrotic effects, and hence could be used to improve the prognosis of DKD patients.^58^

### Redox imbalance in DKD

OS is the first stage of DKD and activates pathological pathways in practically all types of renal cells, including endothelial, mesangial, epithelial, tubular cells and podocytes.^19^ OS results from an imbalance in which the increase in RONs overwhelms the lower efficient (enzymatic and non-enzymatic) antioxidant system, leading to the redox imbalance between pro- and antioxidants.^59,60^

The generation of RONs occurs in many types of cells in the kidneys and infiltrating cells, such as immune cells, neutrophils and macrophages. A substantial increase in glucose auto-oxidation, combined with greater ETC activation and mitochondrial stress, is accompanied by increased RON production, accounting for nearly 80% of all reactive species.^25,61^ In addition to these pathways, other enzymatic systems, such as uncoupled eNOS and NOXs, and non-enzymatic systems, such as Ang II, are involved in ROS generation in the kidney of diabetic patients and obese diabetic patients.^62^

The overproduction of RON induced by hyperglycemia reduces the expression of antioxidant enzymes, including the superoxide dismutase (SOD) (particularly the manganese SOD subtype that acts in the mitochondria), thioredoxin reductase, and catalase (CAT), and decreases regeneration of reduced glutathione (GSH) by activation of the polyol pathway. In addition, spontaneous reduction of non-enzymatic antioxidants occurs in consequence of increased ROS in response to increased demand.^63^ The SOD is considered the main physiological defense against ROS, as it initiates the enzymatic antioxidant defense by reacting with O2^-•^ to form H_2_O_2_, which will be degraded by CAT and GPx (Figure 1).^21,59^

In low concentrations, RONs modulate transcription factors of antioxidant enzymes, essential for OS attenuation. Among these transcription factors, there is the nuclear erythroid 2-related factor 2 (NRF2), which translocate to the nucleus to activate the transcription of genes responsible for codifying antioxidant enzymes like SOD, CAT and GPx, thereby suppressing the NF-kB activity. However, in ROS overproduction, as found in DM, these defenses are not effective in blocking or preventing the establishment of the redox imbalance.^64^

In diabetic kidney, RONs decreases the expression of sirtuins (SIRT), enzymes responsible or modulating the regeneration of antioxidants via acetylation of the ETC, essential for the stimulation of mitochondrial SOD and induction of transcription factors, (such as the PGC1-α), attenuating mitochondrial stress and NRF2 activation.^62^ Also, the O-GlcNAc, product of hexosamines, attenuates the SIRT activity, contributing to exacerbation of this process in DM.^63^ In the kidney of diabetic rats, reduced expression of PGC-1α, a regulator of oxidative metabolism and mitochondrial biogenesis, has been associated with higher production of RONs by aggravating mitochondrial dysfunction and fragmentation.^65^

A gradual decrease in the antioxidant defenses in chronic kidney disease has been described *in vivo*, opening a new field of treatment.^66^ Recent studies have shown the efficacy of high antioxidant intake as an adjuvant in the treatment of DKD, by helping in both enzymatic and non-enzymatic antioxidant defense against harmful compounds.^67^

In a recent meta-analysis, Bolignano et al.^68^ evaluated 14 studies (4,345 participants) for the effects of antioxidant supplementation (including vitamin C, vitamin E and zinc, either combined or alone) on DKD disease progression and markers of renal function. The authors concluded that the antioxidant therapy significantly decreased albuminuria, but apparently had no tangible effect on renal function in patients with diabetic kidney disease. Stronger evidence of benefits was found for vitamin E at doses varying from 480 mg to 1,200 mg/day.

Experimental studies on vitamin and mineral supplementation, such as vitamin D, vitamin E and zinc, have shown favorable results in reducing renal failure, inflammation and OS.^67-70^ Besides, phenolic compounds and flavonoids have shown beneficial effects as therapeutic agents in the treatment of DKD in cells and animals.^71^

### Immunological disorders in DKD: the role of inflammation

DKD has been associated with systemic and intrarenal inflammation. Persistent metabolic and hemodynamic stimuli in diabetic kidney result in cell lesion that releases molecules known DAMPs - danger-associated molecular pattern - including PGAs, ROS, FFAs. These compounds interact with pattern recognition receptors, including Toll-like receptors 2 and 4 and RAGE, positively regulated by hyperglycemia. In the presence of DAMPs/receptors interaction, the intrarenal innate immune response is activated.^5^

The myeloid lineage of innate immune cells causes renal inflammation in diabetic conditions, with involvement of several immune cells in the pathogenesis and severity of renal damage. However, pro-inflammatory factors released in diabetic renal tissue include not only infiltrating inflammatory cells, but also cytokines and chemokines found in non-immune cells, such as in parenchymal cells (podocytes, and endothelial, epithelial, mesangial and tubular cells), exacerbating the inflammatory process that leads to progressive damages in DKD (Chart 1).^19^

**Chart 1:** Inflammatory cytokines and their effects on renal function in diabetes mellitus

Cytokines	Stimulated by	Specialized producing cells	Exerts positive effects on	Effects on DKD	Target cells in the kidneys	Ref.
IL-1α, IL-1β	Inflammasome IL-18 and NF-κB	Macrophages, Granulocytes* Tubular epithelial Endothelial, Mesangial† Fibroblasts‡	↑ ICAM-1, ↑ VCAM-1, ↑ Prostaglandin E2	↑ Intraglomerular hemodynamic abnormality, ↑ Synthesis of hyaluronic acid, ↑ Proliferation of mesangial cells and fibroblasts, ↑ECM accumulation	Epithelial, Mesangial, Tubular	[77, 83]
IL-6	Hyperglycemia, AGEs, TNF-α, LPS, IL-1, IL-4	T lymphocytes, Macrophages, Neutrophils* Endothelial, Podocytes, Mesangial, Tubular epithelial† Fibroblasts‡	↑ MCP-1, ↑ expression of Ang II receptors, ↑ ROS	↑ Recruitment of monocytes, ↑ Differentiation of macrophages, ↑ Synthesis of fibronectin, ↑ Synthesis and accumulation of ECM, ↑ Mesangial cell proliferation, ↑ Endothelial dysfunction ↑ Tubulointerstitial fibrosis	Mesangial, Podocytes, Endothelial, Tubular epithelial	[84, 85]
IL-18	NF-κB, Inflammasome, Caspase-1	T lymphocytes and Macrophages* Epithelial, Tubular†	↑ IFN-γ, ↑ IL-1, IL-6, TNF-α, iNOS, ICAM-1, TGF-β, MCP-1	↑ Apoptosis of endothelial cells, ↑ infiltration off macrophages and neutrophils	Endothelial, Tubular epithelial	[30, 86, 87]
TNF-α	NF-κB	Dendritic, Monocytes, Macrophages, Tlymphocytes* Mesangial, Endothelial, Tubular†	↑ Immune response ↑ NF-κB	↑ Inflammatory cells, cell infiltration ↑ Citotoxicity, apoptosis, ↑ Endothelial permeability, ↓ Capillary wall barrier function, ↑ PKC, ↑NOX, ↑ ROS; ↑ ECM	Mesangial, Podocytes, Endothelial, Glomerular; Tubular epithelial	[19, 78, 83]

* Infiltrating immune cells;

† Renal cells;

‡ Other cell types. Ang II: angiotensin 2; ROS: reactive oxygen species; TNF-a: tumor necrosis factors alfa; NF-κB: nuclear factor-kappa B; IFN-γ: interferon gamma; IL-1: interleukin 1; IL-1a: interleukin 1 alpha; IL-1b: interleukin 1 beta; IL-6: interleukin 6; IL-18: interleukin 18; IL-4:interleukin 4; LPS: lipopolysaccharide; ICAM-1: intercellular adhesion molecule 1; VCAM-1: vascular cell adhesion molecule; ECM: extracellular matrix; NOX: NADPH oxidase; AGE: advanced glycation end-products; MCP-1: monocyte chemoattractant protein; PKC: protein kinase C; iNOS: nitric oxide synthase; TGF-b: transforming growth factor-beta

In addition, the binding of DAMPs with their receptors has been associated with activation of molecular and transcription factors that promote activation of NF-κB, facilitating the expression of many pro-inflammatory genes (cytokines, chemokines, adhesion molecules, immune receptors and growth factors). Consequently, NF-κB has been considered a master regulator of immune responses and inflammation in DKD.^26,72^

The main proinflammatory cytokines are IL-1, IL-6, IL18 and TNF-α; all of them have autocrine, paracrine and juxtacrine mechanisms with pleiotropic effects that regulate the expression of cytokines, interleukins, TNF-α, interferons, growth factors, adhesion molecules and nuclear transcription factors, promoting the increase and perpetuation of inflammation and OS in diabetic kidney (Chart 1).^73,74^

Intracellular metabolic changes with increased AGEs and ROS lead to increased release of MCP-1, which promotes the activation of monocytes and macrophages. These, in turn, are associated with increased expression of adhesion molecules and synthesis of pro-inflammatory cytokines, leading to hyperfiltration and glomerular lesions, typical of DKD.^75,76^

Due to its close relationship with obesity, kidney damage of patients with type 2 DM is associated with early activation of the immune system, which is related to chronic, low-grade systemic inflammation induced by adipose tissue.^44,50^

### Redox imbalance and inflammation in DKD: a vicious circle

**Figure 3:**
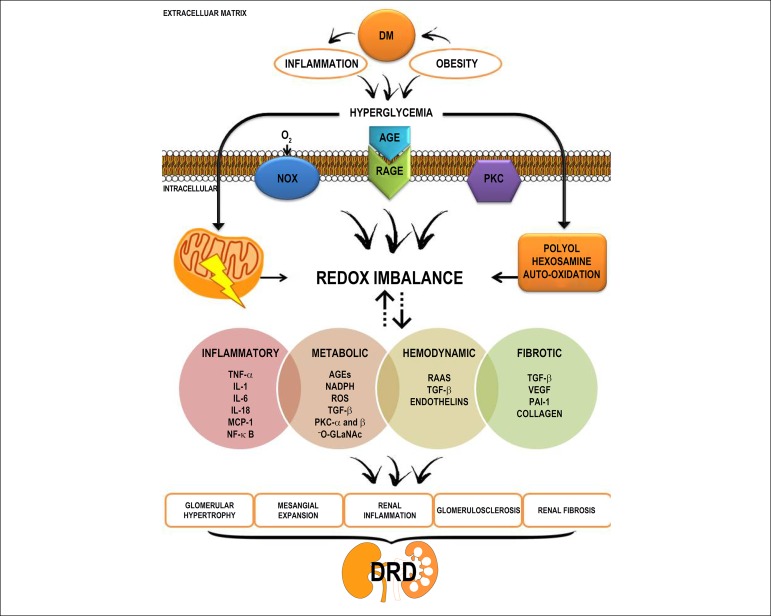
Mediators of kidney injury induced by chronic hyperglycemia via redox imbalance and inflammation in the pathogenesis of diabetic kidney disease. ROS: reactive oxygen species; ERK: extracellular signal-related kinases; TNF- α: tumor necrosis factor alpha; NF-κB: nuclear factor-kappa B; VEGF: the vascular endothelial growth factor; IL-1: interleukin 1; IL-6: interleukin 6; IL-18: interleukin 18; ECM: extracellular matrix; NOX: NADPH oxidase; -O-GLaNAc: O-glycosylated into N-acetylglucosamine; PAI-1: plasminogen activator inhibitor-1; AGE: advanced glycation end-products; PKC: protein kinase C; MCP-1: monocyte chemotactic protein-1; RPGA: receptor for advanced glycation end products; RAAS: renin-angiotensin aldosterone system; TGF-β: transforming growth factor-beta.

Several hemodynamic and metabolic pathways are involved in the pathogenesis of DKD. In a common pathway, the interrelation between redox imbalance and inflammation induced by hyperglycemia occurs by mechanisms that involves cellular and molecular processes in a cascade of bioenergetic changes, promoting changes in extracellular, cellular and mitochondrial morphology, genetic expression modulation, induction of lesions, tissue hypertrophy, and renal fibrosis and necrosis (Figure 3).^74^

Inflammation is mediated by the upregulation of NF-κB expression by OS, AGEs and TNF-α, which controls the immune response by stimulating genetic expression of pro-inflammatory cytokines, adhesion molecules, NOS, cell proliferation and progression of the inflammatory cycle and OS.^77,78^ ROS and the AGE-RAGE interaction, stimulated by DM-related hyperglycemia, act as mediators of the multiprotein complex inflammasome Nlrp, which regulates the cleavage of pro-inflammatory cytokines from the mature, active forms into innate immune cells, renal endothelial cells, glomerular cells and podocytes.^79^

The upregulation of pro-inflammatory cytokines (IL-1, IL-6, IL-18, IFN-γ), mediated by AGE/RAGE, TNF-α and NF-κB, causes an increase in RONs and transcription factors (Chart 1), which lead to local and systemic inflammation, glomerular and tubular lesions, and ultimately, albuminuria.^80^ Among the cytokines, TNF-α is known to cause direct cytotoxicity and apoptosis of renal cells.^17^ A recent meta-analysis showed a statistically significant increase in serum concentrations of TNF-α in type 2 DM patients, especially in those with DKD, suggesting that increased inflammatory load in DKD contributes to disease progression.^81^

The expression of profibrotic transcription factors, such as the TGF-β and connective tissue growth factor, triggers the recruitment of extracellular matrix-producing cells, accelerating renal sclerotic and fibrotic processes.^9^ TGF-β plays pleiotropic effects, promoting hyperplasia and hypertrophy of renal cells. In the extracellular matrix, TGF-β is found in a latent form, bound to proteins, requiring cleavage to release of its free, active form. This activation is performed by mediators produced under hyperglycemic condition, including AGEs, ROS, DAG, PKC, Ang II, among others. Once activated, TGF-β binds to its cell receptor, and regulates the transcription of target genes, including collagen types I, III and IV, fibronectin, plasminogen, and PAI-I, with net effect of protein synthesis and expansion of the extracellular matrix, glomerulosclerosis and renal fibrosis. It also activates NF-κB, contributing to the production of proinflammatory cytokines, exacerbating local inflammation.^34,74,82^

## Conclusion

Recently, there has been increasing evidence that redox imbalance and inflammation in response to intermittent or chronic exposure to hyperglycemia play an important role in initiation and perpetuation of DM complications, including DKD. They are now considered the main contributors to the development of DKD and end-stage kidney disease. New pathological pathways, associated with renal dysfunction in DM and that particularly exacerbate metabolic pathways, have been identified, such as the association between DKD and obesity. Therefore, metabolic, inflammatory and oxidative interference of DM and other risk factors for DKD should be continuously investigated and updated not only to improve the understanding of the mechanisms, but also to determine new therapeutic targets.
